# Toxic Tales—Recent Histories of Pollution, Poisoning, and Pesticides (ca. 1800–2010)

**DOI:** 10.1007/s00048-018-0190-2

**Published:** 2018-04-27

**Authors:** Claas Kirchhelle

**Affiliations:** Wellcome Unit for the History of Medicine, Oxford Martin School, University of Oxford, Oxford, UK

**David Arnold 2016: *Toxic Histories. Poison and Pollution in Modern India***. Cambridge: Cambridge University Press, hardback, 241 p., £34.99, ISBN 978-1-107-12697-8.

**Michelle Mart 2015: *Pesticides, a Love Story: America’s Enduring Embrace of Dangerous Chemicals***. Lawrence: University Press of Kansas, hardback, 337 p., $ 34.95, ISBN 978-0-7006-2128-6.

**Richard S. Newman 2016: *Love Canal: A Toxic History from Colonial Times to the Present***. New York: Oxford University Press, hardback, 306 p., $29.95, ISBN 978-0-19-537483-4.

The past two decades have seen a surge of publications on the histories of various toxic substances. Often fusing approaches from environmental history and the histories of science, medicine, and technology, historians have explored the manufacturing of hazardous products and by-products; the various uses and cultural perceptions of toxic substances; their impact on health and the environment; and attempts to regulate toxic risk. This review summarizes major themes arising from the growing body of historical work on the ‘toxic’. In synthesizing these themes, the review highlights a common ‘toxic chronology’ that emerges from existing literature and discusses three new monographs in relation to gaps and weaknesses identified in current research.

## A Toxic Chronology

In 2016, José Ramón Bertomeu-Sánchez and Ximo Guillem-Llobat discussed recent literature on ‘poisons in society and culture’. Their thematic overview ordered historiography along the lines of crime, food, health, environment, experts and activists, spaces, and uncertainties (Bertomeu-Sánchez & Guillem-Llobat 2016). While the authors highlighted the breadth of historiography, this review attempts a more concise synthesis of existing literature. It argues, what emerges from several decades of research is a common chronology of the toxic. Focussing on the period from the mid-eighteenth century onwards, historians have established four broad phases of human-toxic interactions: (1) a period of normalising toxic exposure; (2) a period of attempting to curb toxic hazards with technical fixes; (3) a period of coming to appreciate toxicity’s environmental dimensions; and (4) a period of fragmenting public and expert understandings of the toxic, a realisation of the unequal burdens of toxicity, and of coming to terms with permanent toxic exposure.

### Normalisation

The first phase of this ‘toxic chronology’ can be characterised as an era of normalisation. Focussing on France and Britain, Thomas Le Roux, Jean-Baptiste Fressoz, and Peter Thorsheim have described how the period around 1800 saw a young generation of planners, industrialists, and experts dismantle *ancien regime *complaints and zoning mechanisms. A reformed medical-legal discourse on toxicity subsequently ‘normalised’ the presence of large new polluting industries in urban and rural communities and indemnified owners against law suits resulting from damage to property and health (Thorsheim 2006; Le Roux 2011; Fressoz & Le Roux 2011). Focussing on air pollution, Frank Uekötter has described how the logic of normalising toxic risk continued throughout the nineteenth and early twentieth centuries. With industrial output and urban populations growing rapidly, municipal and national authorities had to adjudicate between citizen complaints and commercial interests. While smoking chimneys and dead rivers were often reinterpreted as signs of progress, specific groups of experts like social hygienists and civil engineers were tasked with devising technical solutions like more efficient boilers, ‘clean’ coal, higher chimneys, or hydrological interventions into local and regional environments to alleviate the most visible forms of pollution (Uekötter 2009). In some cases, particularly controversial practices like the open-air roasting of ores were banned (Guillem-Llobat 2017).

Toxic substances not only spread as a result of industrial pollution but also because of public demand. Historians like Judith Rainhorn and Carolyn Cobbold have highlighted how well-known toxic substances like arsenic, lead, and mercury and new synthetic coal tar dyes entered homes and bodies via wallpapers, paint, cosmetics, and dyed food (Rainhorn 2013; Cobbold 2016; ongoing research by Amélie Müller). Following the logic of the dose makes the poison, medicine was another source of toxic exposure. For much of the eighteenth and nineteenth centuries, physicians and other practitioners resorted to both vegetable and mineral ‘poisons’ to treat physical and emotional maladies (Harrison 2010; Arnold 2016; ongoing work on mercury by Andrew Cunningham). As Katherine Watson and others have highlighted, toxic substances also acquired a darker prominence as poisons. The subversive nature of poisons in the hands of women, slaves, and colonial subjects triggered poison panics and the development of forensic toxicology and medical jurisprudence (Watson 2004; Savage 2007; Bertomeu-Sánchez 2016).

### Fixing Toxicity

From the late nineteenth century onwards, a growing number of experts, politicians, consumers, unionists, and progressive industrialists attempted to measure and devise safe boundaries within which toxic practices could play out.

In the case of food, historians have explored how concerns about chemical adulteration and the new discipline of bacteriology led to investigations into the safety of synthetic additives and sweeteners, inspections for microbial and chemical contamination as well as pasteurisation and hygiene requirements (Hardy 2015; Atkins 2010; Smith & Philips 2000; Guillem-Llobat 2012). In medicine, new concepts of toxicants and toxins influenced the development of serum therapy and antitoxins. Meanwhile, the early era of chemotherapy saw already familiar toxic substances like arsenic and new synthetic substances such as sulphonamides turn into ‘magic bullets’ for the treatment of microbial infection and later for cancer. Growing concerns about narcotics and toxic side-effects gradually led to the creation of prescription-only restrictions for certain substance groups. In some countries, the interwar period saw new regulatory bodies like the Food and Drug Administration (FDA) tasked with assessing products’ toxic risks ahead of licensing (Anderson 2008; Carpenter 2010).

Occupational exposure to toxic substances turned into a further area of concern. Described by Christopher Sellers, Frederick Rowe Davis, and others, the period around 1900 saw a new generation of toxicologists, industrial hygienists, and other “measuring scientists” (Schwerin 2009: 7) focus not only on acute toxicity but also on low-dose and long-term exposure to substances like lead and new hazards like radiation (Sellers 1997; Sellers & Melling 2012; Davis 2014). Resulting risk scenarios led to attempts in differentiating between acceptable and inacceptable exposure. Coinciding with a shift of mortality away from infectious disease to cancer and heart disease and contemporary concerns about racial degeneration, the creation of acceptable boundary values of exposure was strongly impacted by economic interests, divergent understandings of toxicity, and competing professional interests (Reinhardt 2008; Proctor 2000). Although criticism of artificial substances and lifestyles had grown steadily since the *fin de siècle*, public criticism of toxic exposure rarely resulted in a full rejection of industrial progress but in a quest to make exposure safe.

### Toxic Environments

Trust in technical fixes of toxic hazards began to erode in the decades following the end of the Second World War. Historians like Edmund Russell, Linda Nash, Nathalie Jas, and Kendra Smith-Howard have explored the gradual environmental shift of toxicity concerns. Whereas public discussions of toxic exposure had previously been limited to individual sites, practices, and products, the post-war era saw toxic fears encompass not only local but also regional and global environments. The environmental shift of toxicity concerns was accompanied by demands to protect both natural wilderness and vernacular landscapes like homes, gardens, and cities (Russell 2001; Schwerin 2008; Jas 2007; Nash 2008; Smith-Howard 2013). Coinciding with a new medical focus on risk factors and preventive health care (Lengwiler & Madarász 2010; Timmermann 2010), public awareness for the toxic interconnectedness of humans with their environment was heightened by scares about chemically contaminated food and bodies and radioactive fallout. In Europe and North America, demands for ‘pure’ food and environments led to a rapid expansion of what Sheila Jasanoff (1994) has termed regulatory science. Scientists and regulators were once again expected to mediate between health concerns and the post-war boom of industrial production, which saw a flood of new substances inundate the global market. While officials frequently established tolerances for ‘safe’ exposure to hazardous substances, cultural taboos, growing cancer fears, and new research on mutagenicity led to demands for zero-tolerance of toxic and carcinogenic substances in food and nuclear testing bans—non-toxic and non-carcinogenic substances escaped public criticism (Burkett 2012; Chadarevian 2006; Gaudillière 2010; Jas 2013; Kirchhelle 2016). Described by Alan Marcus (1994), Heiko Stoff (2015), and Nancy Langston (2010), the most potent legislative expressions of zero-tolerance demands were the passage of the 1958 Delaney Clause in the USA and West Germany’s 1958 Food Law respectively.

With powerful links forming between concerned scientists as well as consumer and environmentalist activists, the 1960s saw tensions over toxic exposure reach fever pitch. Regulators and manufacturers were forced to respond to new scientific warnings about toxic exposure as well as media pressure and a series of critical international bestsellers—most notably Rachel Carson’s 1962 *Silent Spring*. Ensuing controversies over DDT and DES and the thalidomide scandal further undermined public trust in official safety claims. US and European governments reacted to public pressure by creating new environmental agencies, strengthening regulatory scientists, and passing stricter substance regulations. Experienced in defending low-dose exposure to known hazards like lead since the turn of the century (Markowitz & Rosner 2002), industrial actors often responded to attempts of banning substances with counter science. As described by Robert Proctor (2008), Naomi Oreskes and Eric Conway (2010), lobbyists often received support from an older generation of scientists whose defence of controversial technologies was motivated by Neomalthusian scenarios of overpopulation, and a developmentalist anti-communist agenda. Older scientists also rejected the often left-leaning politics of younger critical scientists. Over time, competing regulatory, environmentalist, and industrial expertise undermined the public authority of science and politicised environmentalist discourse. Outside of Europe and the US, environmentalist regulations and values often developed differently. Need-based environmental values—“the environmentalism of the poor” (Guha & Alier 1997)—could diverge substantially from those of middle-class Westerners and did not necessarily evoke a wider public rejection of known toxic hazards. Meanwhile, substances like DDT, which had been banned in the West, continued to be legally exported to other parts of the world or were produced there directly.

### Fragmentation

Whereas earlier protest against toxic exposure had often drawn on support from all sides of the political spectrum, the 1970s saw environmentalist movements become politicised in a number of countries and lead to the founding of Green parties. Meanwhile, the precautionary era of US substance regulation that had culminated in the 1958 Delaney Clause and 1972 DDT ban stalled at the very moment that European integration was fostering precautionary regulations on the other side of the Atlantic. While European regulators restricted hormonal growth promoters, tried to curb acid rain, and introduced stricter industry reporting standards, their US counterparts struggled to ban toxic and carcinogenic drugs, pesticides, and sweeteners. Benefiting from complicated proof of harm requirements, an emphasis on economic cost-benefit analyses, and new testing methodologies, US regulation critics not only stalled official action but also began to rollback existing regulations under the Reagan Administration (Uekötter 2011; Vogel 2012; Boudia 2014; Creager 2014).

Despite growing regulatory gaps, popular environmentalist values became increasingly international. Since the 1970s, disasters and scandals such as Seveso (1976), Love Canal (1978), Bhopal (1984), and Chernobyl (1986) heightened public awareness of global toxic hazards and the long-term costs of contaminated landscapes. Protest against toxic hazards also became international. While environmental justice movements highlighted the plight of disadvantaged communities living with toxic burdens, major NGOs like Greenpeace began to stage anti-pollution protests like the occupation of the Brent Spar oil platform in 1995. Protests were not limited to the West. In countries like China and India, middle classes have become increasingly vocal in their demands for ‘non-toxic’ environments while Latin American farm laborers and migrant workers have protested against their occupational exposure to toxic substances (Wöbse 2004; Uekötter & Kirchhelle 2012; Bohme 2015).

Since the 1980s, the prospect of biotechnology and research on endocrine disruptors have further added to the list of international toxic concerns and challenged traditional dose-response and carcinogenicity-oriented risk regulation. However, despite the mainstreaming of environmentalist values, definitions over what it means to be Green, and what risks are acceptable continue to divide regulators and the general public. In Europe, the 1996 BSE crisis and the EU’s official adoption of the precautionary principle in 2000 led to bans of GMOs and other substances (Lezaun 2004; Lezaun & Schneider 2012). By contrast, Sarah Vogel has described how US regulators simultaneously dismantled vestiges of precautionary zero-tolerance regulation (Vogel 2013). While the spread of GMOs and agricultural monocultures is currently facilitating a further global increase of pesticide and herbicide use, private market solutions to perceived toxic risks have also proliferated. Offering allegedly pure food and guarantees of personal safety on an increasingly industrial scale since the 1970s, the success of organic producers is in part a market-driven response to regulators’ failure to ease toxic anxieties (O’Sullivan 2015).

## Research Challenges

The described chronology of normalisation, technical fixes, environmental awareness, and fragmentation testifies to the wealth of ‘toxic historiography’. Historians have reconstructed the origins and path dependencies of our toxic infrastructures, the challenges of regulation, and divergent cultural risk perceptions. In doing so, they have highlighted the importance of ‘boundary work’ and the complex trade-offs between public anxieties, economic interests, and scientific knowledge that underpin regulations and shape toxic realities. Historians have moreover played an important role in raising awareness for environmental injustice and holding states and polluters to account. Over the past decade, researchers like Dominque Pestre, Soraya Boudia, Nathalie Jas, and Sheila Jasanoff have also engaged policy makers and challenged simplistic economic and regulatory short-termism (Boudia & Jas 2014; Jasanoff 2007; Pestre 2008).

However, existing toxic literature also has a number of weaknesses. On the one hand, its wide scope can lead to confusion. Industrial pollutants, radioactivity, chemical and microbial food poisoning, self-intoxication, and the use of poisons in medical and criminal settings are often presented as ‘toxic’ without differentiating sufficiently between substances’ unique qualities and histories. On the other hand, there has also been a tendency to artificially distinguish different forms of toxic contamination—especially when it comes to the chemical and biological contamination of food. Many accounts also do not focus sufficiently on the complex cultural and scientific genesis—or loss—of toxic assignations. Studying and comparing the cultural and scientific origins of toxic assignations and assignations’ effects on evaluations of other technologies could be a rewarding way to expand the analytical breadth of current toxic historiography.

Broadening the geographic focus of current research is also desirable. Perhaps reflecting the origins and strength of environmental history in the US, many recent books focus on toxic hazards only in the American context. In doing so, they follow a narrative of toxic proliferation, outrage, and limited reform that matches accounts of US environmentalism. This limited geographic focus not only neglects toxic knowledge and interactions with other parts of the world, but also runs danger of overestimating the impact of US environmentalist icons like Rachel Carson outside of the Anglophone world. There is moreover a tendency to contrast perceived failures of US regulation following the neoliberal turn with an allegedly better precautionary reality in Europe. Such a narrative runs danger of ignoring problems within Europe, insufficiently differentiating between different European nation states, and downplaying other contingent factors influencing US and European substance regulation. Similar problems with narrow national narratives also characterise European toxic histories. Looking East of the Iron Curtain and studying Japan, India, and South America, a limited number of publications is beginning to broaden our understanding of international toxic histories (such as Walker 2010; Brown 2013; Bohme 2015). However, for truly global histories to emerge, historians will have to trace the spread of toxic substances, knowledge, and regulation across national borders and continents. Although many archives remain closed, histories of the international companies producing and selling toxic products could be one way of achieving this goal. Another way of studying varying definitions of the ‘toxic’ might be to look at the international proliferation of ‘pure’ organic food and organic standards and supply chains.

Perhaps the most significant challenge that remains for historians is moving beyond standard tales of problem identification and remediation. Many land- and seascapes will remain contaminated with radiation or toxic hazards long after initial problems have been identified and pollutants banned. The impact on communities living in or dependent on these areas is not well understood. By studying current and historic ‘toxic’ communities, historians can play an important role in challenging simplistic narratives of toxic redemption and provide insights into societies’ ability or failure to adapt to permanent toxic exposure.

## New Research

Three recent publications have addressed the described research challenges by not only extending the thematic and geographical scope of toxic histories but also by focusing on life in contaminated landscapes.

Published in 2016, David Arnold’s* Toxic Histories. Poisons and Pollution in India* studies the social and scientific cognition of poisons in pre-colonial, colonial, and independent India. Arnold’s aim is to study “toxicity as an overarching concept” (p. 10) of Indian history that was not grounded in substances’ “objective reality or discreet materiality” (p. 9) but evolved as a result of interlayered cultural, scientific, and regulatory practices and discourse. He proposes the existence of a “toxic continuum” (p. 11) connecting modern concepts of pollution with earlier notions of poisoning. Exploring the ‘social life’ of poisons in pre-colonial India, Arnold analyses mythological accounts of vegetable, mineral, and snake poisons, poisons’ use in Unani-tipp and Ayurvedic medicine, their use as aphrodisiacs, for abortion, infanticide, and suicide, and cases of accidental poisoning caused by famine foods. Arnold next reconstructs how India’s toxic environment was affected by empire. Whereas pre-colonial poison culture was fragmented, the colonial lens evaluated and ordered substances from the perspective of economic and therapeutic usefulness but also as potential hazards due to medical or criminal misuse. Emerging alongside the rise of a more formal empire from the 1830s onwards, an imperial pharmakon combined British and Indian poison cultures. The ambiguities of this pharmakon allowed Indian experts to mediate between native and colonial toxic knowledge systems and enabled substances like arsenic to be simultaneously identified as a dangerous poison and a useful industrial substance.

In the “intimate” (p. 130) spatial settings of imperialism, poisons could also challenge the established order. Arnold reconstructs how ‘poison panics’ about criminal cults, cattle poisoning, and alleged poisonings of British officials fostered the rise of forensic toxicology and medical jurisprudence from the 1850s onwards. In contrast to British poison fears, Indian concerns focused mostly on contaminated food and water and ritual pollution. Reacting to poison and pollution concerns, British administrators restored “epistemic authority” (p. 210) by establishing chemical—and later bacteriological—surveillance systems and creating taxonomies for vegetable poisons. The 1904 Indian Poisons Act’s restriction of access to substances like arsenic marked an important transition from person-centered fears of poisoning to wider concerns about contaminated foods, unregulated drug sales, and polluted urban environments. Fears of pollution were not just an imperial preoccupation but also shared by India’s rising middle classes. Both parties saw toxic substances as a hazard and solution to urban problems. Arnold illustrates this nuanced view of toxicity by studying the simultaneous use of poisons to curb disease and animal pests and often half-hearted attempts to contain toxic hazards in the workplace and in urban air, water, and food. Attempts to use toxicity whilst containing its hazards are ongoing. After Independence, India’s “Manichean” (p. 16) relationship with poisons continued in the form of the mass-import and mass-production of pesticides during the Green Revolution and parallel problems with contaminated landscapes, agricultural dependency, and the 1984 Bhopal catastrophe.

Arnold’s book offers a fascinating glimpse into India’s ‘toxic’ history. It sheds light on how ‘poisons’ were constituted by often divergent cultural understandings, economic imperatives, and scientific and regulatory inquiry. Combining accounts of pollution, the opium and arsenic trade, famine foods as well as famous Indian and British murder cases, the book makes an interesting case for a ‘toxic continuum’ between past understandings of poisons and current perceptions of pollution.

However, there are also a number of issues with Arnold’s occasionally rather abrupt narrative. For one, it remains questionable whether the reader is really encountering an Indian or an imperial history of poisoning. The book’s narrative jumps between ancient history, the colonial period, and Bhopal. Despite analysing the consumption of famine foods, its voices are often those of colonial officials and Indian elites. Meanwhile, readers learn little about the development of toxic and chemical sciences in the British metropolis, how poisons were measured and tolerances were established, and how Indian toxic expertise developed after Independence (Pakistan is not discussed at all). Readers may also ask whether poison panics drove the establishment of imperial toxicology or whether the colonial lens ‘created’ a ‘poison culture’ by establishing a toxic epidemiology in the first place.

With the book itself admitting that ‘poison scares’ often had a limited impact, it also remains questionable whether the described episodes constitute a national discourse of toxicity, and whether episodes like the Agra murders during which a pair of Anglo-Indian lovers killed their respective partners in 1911/1912 indicated a pending crisis of imperialism (p. 139–142). Another question is whether contemporaries really grouped microbial and chemical food contamination, snake bites, criminal poisoning, and air, water, and ritual pollution under the same ‘toxic’ umbrella? While Arnold claims that old fears of poisoning were subsumed into “a new language of toxicity, adulteration, contamination and pollution” (p. 164), the book does not study discontinuities within this process—especially regarding differences in the perception of chemical and bacterial toxins or of new synthetic pesticides and older poisons. Given the book’s chronological leaps after 1947, the ‘toxic continuum’ connecting Bhopal with nineteenth century poison murder at times seems porous.

Also published in 2016, Richard Newman’s *Love Canal: A Toxic History From Colonial Times To The Present* (2016) is a local “environmental epic” (p. 9) of America’s Buffalo-Niagara region and of Love Canal as “the world’s most famous toxic trash heap.” (p. 2) The first part of this eminently readable book spans the Niagara landscape’s use by Native Americans to its transformation into one of America’s industrial powerhouses. Focusing on Europeans’ view of landscape as a “usable, and even disposable, commodity,” (p. 18) Newman studies various regional development schemes from the seventeenth century onwards. One of these schemes was entrepreneur William Love’s vision of a gigantic hydroelectricity plant and model city close to the Niagara Falls. Although Love’s scheme came to nothing, the earthworks for a power canal remained. By the 1940s, Love’s canal had been purchased by Hooker Chemicals. Located in Niagara Falls since 1905, Hooker produced chemicals ranging from caustic soda to synthetic pesticides, polyester resins, and PVC. Having exhausted on-site storage, Hooker dumped accruing waste in Love Canal. Once it was filled, Love Canal was covered with earth and purchased by the city of Niagara Falls in 1953 despite warnings about the waste. For Newman, lacking concern about the toxic site was indicative of contemporary trust in a clean ‘synthetic future’ and of concepts of humans as divorced from their immediate environment.

The second part of Newman’s book examines Love Canal’s transformation into part of the suburban American dream—and into a toxic nightmare for its residents. After initially ignoring health problems, miscarriages, and birth defects, the mid-1970s saw officials acknowledge Love Canal’s toxic nature. By 1978, the failure of *ad hoc* fixes and experts’ confirmation of health hazards led to public disquiet. In August 1978, President Carter declared Love Canal a national emergency—marking the first time this was done for a human-made disaster—and granted funds for the evacuation of a limited number of families and remediation work. Residents left behind in this toxic landscape responded by conducting popular epidemiology and an escalating series of protests. Newman traces how local activists lobbied for a new concept of landscape stewardship that not only encompassed spectacular natural sites like Niagara Falls but also vernacular landscapes like suburbia. Despite internal divisions, grassroots protests attracted national media and political attention and triggered the evacuation of the entire neighborhood in May 1980. According to Newman, Love Canal was a “toxic tipping point” (p. 195) in US history: vernacular landscapes became sites of environmental intervention and residents highlighted the power of grassroots activism.

The third part of Newman’s book is the most innovative. Tracing the history of Love Canal after residents left, it analyses resulting political reforms, attempts to remediate and resettle the site, and controversies over how to remember the toxic tragedy. Responding to Love Canal, US legislators created a superfund for toxic remediation, new toxic waste guidelines, and a national inventory and priorities list for toxic remediation. The superfund was reauthorized in 1986 and included the US’ first right-to-know provisions for chemical substances. Newman also studies the fate of former Love Canal residents. Despite their evacuation, dispersed residents continued to suffer emotionally and physically and to campaign against toxic hazards. Meanwhile, the actual Love Canal site underwent a significant transformation. Newman traces the battles between activists, officials, and environmental engineers over how to contain Love Canal’s toxic hazards and whether it was safe for resettlement. Despite the construction of a sophisticated drainage system and a new cap for the 20,000 tons of toxic sludge, it was clear that Love Canal would require constant intervention to keep its hazards contained. Using new criteria for habitability, city and state officials, however, declared Love Canal fit for resettlement in 1988. Since then, remediation and resettlement have turned Love Canal into a contested memory site. Officially removed from the EPA’s National Priorities List in 2004, many current residents do not want Love Canal activists protesting in their neighborhood, now renamed Blackcreek Village. Although a Love Canal memorial has been placed on the site, it does not celebrate grassroots activism. However, with Blackcreek Village residents recently taking legal action over birth defects and other health problems, battles over Love Canal’s meaning and remediation continue.

Newman’s *longue durée* study of the Niagara landscape breaks with the crisis-oriented narratives of many toxic histories. His book makes a compelling argument for a 1970s environmental reinterpretation of vernacular landscapes, and highlights how definitions of acceptable and non-acceptable hazards were mutually constructed by journalists, bureaucrats, scientists, industrialists, and citizens. The result is a powerful story of Love Canal as a contested memory site for the rise of US grassroots activism, ambitious attempts to remediate toxic landscapes, and the unclear fate of communities living in these landscapes. While it is perhaps excessive to expect yet more detail, missing at times from Newman’s broad analysis is a discussion of other contemporary crises. Surprisingly, Newman does not mention the 1976 Seveso disaster, during which an explosion in a chemical plant contaminated Northern Italian landscapes and people with dioxin. Seveso triggered evacuations, the creation of European toxic inventories, new industry reporting guidelines, and decades of remediation. There are significant parallels between the two crises and Love Canal residents and US politicians must have been aware of the Seveso precedent. A brief description of other toxic incidents could also have provided a useful frame of reference with which to contextualize the mixed outcome of remediation and Love Canal’s impact beyond the US. Newman also does not explore how hazards were measured and whether certain wastes were considered more dangerous than others. This is surprising given the contemporary boom of research on toxic and mutagenic hazards and the shift towards cost-benefit analysis.

Published in 2015, Michelle Mart’s *Pesticides, a Love Story* (2015) asks why rising environmentalist awareness did not reduce US pesticide use. Studying pesticide discourse in the mainstream media, scientific journals, political, and activist literature, the book first explores the post-war mass adoption of three new classes of pesticides and herbicides—the organochlorides, organophosphates, and phenoxy herbicides. According to Mart, the close relationship between media, government, and business induced a conservative mode of journalism that stressed the benefits of new technologies and downplayed hazards. Proving popular amongst homeowners and farmers, pesticide use was also actively promoted by the United States Department of Agriculture (USDA), which asserted that pesticides were safe if users adhered to labels and guidelines. Chemical optimism only gradually waned in light of concerns about tissue build-up, aggressive USDA spraying campaigns, and heightened residue fears in the wake of the 1958 Delaney Clause’s zero-tolerance provision for carcinogens and the 1959 Cranberry Scare. Although Rachel Carson’s *Silent Spring *(1962) catalyzed a cultural ‘breakup’ with pesticides, Mart notes that Carson and other early reformers were very cautious and called only for limited and rational pesticide use rather than wider bans. Culminating in the 1972 DDT ban, this strategy led to significant legislative reform but failed to reduce overall pesticide use.

Mart explores this paradox of rising scepticism and rising use by pointing to how pesticides and herbicides were “ennobled” (p. 83) as part of the Green Revolution, their use in Vietnam, and the public’s tendency to problematize individual aspects rather than overall chemical use. At the domestic level, the 1970s saw the foundation of the Environmental Protection Agency (EPA), the passage of pesticide and chemical legislation, and a new focus on integrated pest management result in stricter premarket testing and residue controls as well as a rhetorical shift away from area spraying. However, despite contemporary disasters like Love Canal and Bhopal, there was no powerful challenge to overall pesticide use. Instead, Americans were satisfied with bans of the most dangerous substances and allegedly higher domestic safety standards. While the use of remaining pesticides was thus culturally normalized, the Reagan years saw limited regulatory safeguards and EPA competencies shrink as a result of a new emphasis on cost-benefit assessments, ‘good science’ (that is, insistence on concrete proof of harm rather than precautionary reasoning), and budgetary cuts.

Since the 1980s, scientists have cautioned that current dose-response and cancer-focused regulations do not adequately capture the long-term hazards of pesticides and endocrine disruptors. Although the growing market for organic food has allowed consumers to opt out of chemical agriculture, it has also produced a false sense of security and fragmented political pressure for pesticide reform. Meanwhile, global pesticide use has rapidly expanded alongside the spread of GMO monocultures. Mart explains the uneasy coexistence of expanded pesticide use and private pesticide wariness by highlighting Americans’ “four bedrock assumptions” about pesticide regulation:

[…] modern human society could, to some degree, manipulate or control the environment; short-term interests were more important than long-term ones; individual and human concerns trumped collective and environmental ones; and environmental decisions should be made on the basis of clear evidence of what had happened, not out of fear for what might happen. (p. 222)

Unfortunately, this cost/benefit lens was often “wholly incompatible with nonquantifiable values such as human health and a clean environment.” (p. 181).

Mart’s book is a *tour de force* of Americans’ relationship with pesticides, herbicides, and other toxic technologies. Although many aspects of the book have been covered by other authors, Mart’s skilful interweaving of various stories provides an innovative and insightful analysis of the paradoxical parallelism of US pesticide wariness and embrace. While making an important contribution to the history of toxic substances, Mart’s book at times runs danger of an ideal type contrast between a rather sinister US cost-benefit psyche and a more far-sighted European precautionary mentality. A closer look at the protectionist motives and actual outcomes of European policy-making may well complicate this narrative. Mart’s focus on the post-war years also ignores pre-war continuities of industrial and organic agriculture. The mixed chronological and thematic organization of the book can be confusing: why is Bhopal discussed prior to Love Canal? Meanwhile, the perspective of actual pesticide users (that is, farmers or homeowners) is lacking. Given the plethora of other perspectives discussed in the book, this omission is surprising. While Mart’s *Love Story* is a horror story of path dependencies and ‘blind love’, her criticism of US ‘pesticide lovers’ is perhaps overly harsh: for inhabitants of a toxic world, conceiving of a non-toxic alternative may well stretch the imagination.
